# The dependence of expression of NF-κB-dependent genes: statistics and evolutionary conservation of control sequences in the promoter and in the 3′ UTR

**DOI:** 10.1186/1471-2164-13-182

**Published:** 2012-05-11

**Authors:** Marta Iwanaszko, Allan R Brasier, Marek Kimmel

**Affiliations:** 1 Institute of Automatic Control, Silesian University of Technology, Akademicka 16, Gliwice, Poland; 2 Department of Medicine, UTMB, 301 University Boulevard, Galveston, USA; 3 Department of Statistics, Rice University, 6100 Main St., Houston, TX, 77005, USA

## Abstract

**Background:**

The NF-κB family plays a prominent role in the innate immune response, cell cycle activation or cell apoptosis. Upon stimulation by pathogen-associated patterns, such as viral RNA a kinase cascade is activated, which strips the NF-κB of its inhibitor IκBα molecule and allows it to translocate into the nucleus. Once in the nucleus, it activates transcription of approximately 90 genes whose kinetics of expression differ relative to when NF-κB translocates into the nucleus, referred to as Early, Middle and Late genes. It is not obvious what mechanism is responsible for segregation of the genes’ timing of transcriptional response.

**Results:**

It is likely that the differences in timing are due, in part, to the number and type of transcription factor binding sites (TFBS), required for NF-κB itself as well as for the putative cofactors, in the Early versus Late genes. We therefore applied an evolutionary analysis of conserved TFBS. We also examined whether transcription dynamic was related to the presence of AU-rich elements (ARE) located in 3′UTR of the mRNA because recent studies have shown that the presence of AREs is associated with rapid gene induction. We found that Early genes were significantly enriched in NF-κB binding sites occurring in evolutionarily conserved domains compared to genes in the Late group. We also found that Early genes had significantly greater number of ARE sequences in the 3′UTR of the gene. The similarities observed among the Early genes were seen in comparison with distant species, while the Late genes promoter regions were much more diversified. Based on the promoter structure and ARE content, Middle genes can be divided into two subgroups which show similarities to Early and Late genes respectively.

**Conclusions:**

Our data suggests that the rapid response of the NF-κB dependent Early genes may be due to both increased gene transcription due to NF-κB loading as well as the contribution of mRNA instability to the transcript profiles. Wider phylogenetic analysis of NF-κB dependent genes provides insight into the degree of cross-species similarity found in the Early genes, opposed to many differences in promoter structure that can be found among the Late genes. These data suggest that activation and expression of the Late genes is much more species-specific than of the Early genes.

## Background

### NF-κB signalling pathway and classification of NF-κB-dependent genes

NF-κB is a family of transcription factors [1] that plays a prominent role in innate immune response among other cellular processes, as reviewed in Tian et al. 2005; [2]. The dynamics of NF-κB translocation has been studied both experimentally and using mathematical and computer modelling [3-6]. Upon stimulation by pathogen-associated molecular patterns, such as viral RNA, a kinase cascade is activated, which eventually strips the NF-κB of its inhibitor IκBα molecule and allows it to translocate into the nucleus. In the nucleus, NF-κB binds to specific palindromic sequences in the regulatory sequences of promoters to activate the transcription of a number of genes approximately 90, of which 74 were systematically examined; [2]. Inspection of the mRNA transcript profiles has further shown that the NF-κB-dependent genes can be categorized by the timing of their activation relative to NF-κB’s translocation into the nucleus [2]. Notably, the Early genes’ peak response occurs at about 30–60 min. after NF-κB translocation, as opposed to the Middle genes’ response at about 3 hrs. and the Late genes’ response at up to 6 hrs. Interestingly, these categories encode distinct molecular functions, the Early genes being predominantly cytokines, Late genes encoding cell surface adhesion molecules and signalling adapter molecules and Middle genes overlapping Late genes’ functions in control of signalling molecules and expression of cell-surface receptors.

It is not obvious what mechanism is responsible for segregation of the genes’ timing of transcriptional response. One likely hypothesis might be that the later the gene is the more cofactors are required to activate it. This hypothesis gave rise to a mathematical model in Paszek et al. [7]. Another hypothesis is that NF-κB has to be primed by a post-translational modification such as amino acid-specific phosphorylation or acetylation to act as a transcription factor for a given gene, and that such processing requires additional time in some cases. This latter hypothesis was in part confirmed by Nowak et al. [8].

The question we address here is how gene’s expression is regulated by transcription factor binding sites (TFBS) in the gene’s promoter. NF-κB family is sequence specific, with four identified binding motifs corresponding to different family members. Identified binding motifs are 10 nucleotides long (except for the motive for the heterodimeric particle) and have a characteristic guanine triplet (GGG) opening the motif. Given this, it seems correct to use the software finding TFBS in genetic sequences. Devising software specialized in searching for regulatory elements in noncoding regions of the genome is a serious challenge, but advances in gene expression analysis technologies allow detection using computational TFBS methods and development of databases containing position weight matrices (PWMs), such as JASPAR [9] and TRANSFAC [10]. Analysis of sequences for the presence of known TFBS by using PWMs only can produce a large number of false positive predictions; therefore computational TFBS detection must be enriched with some other methods helping find functionally relevant TFBS [11]. This can be accomplished using phylogenetic footprinting, which is based on the assumption that TFBS should be highly conserved in comparison to non-regulatory regions close to genes [12]. This approach is used by ConSite [13], which uses ORCA algorithm [14] for phylogenetic footprinting and JASPAR database for TFBS sequence identification. Recent research suggests that in transcriptional regulatory regions modules occur, which contain clusters of TFBS [15] that can be distinguished from non-regulatory areas by high conservation.

Regulation of mRNA degradation is an important mechanism controlling the gene expression profile. The AU-rich element (ARE) is a 50–150 nucleotides long region containing high number of adenine and uridine bases in the 3′UTR of the mRNA transcript [16]. AREs role is to target mRNA for rapid degradation; they are usually found in mRNA of cytokines, transcription factors, and cell growth proteins. It has been estimated that 5% - 8% of the human genome encodes ARE-containing mRNAs [17]. Although the AREs are pleomorphic, studies have identified the AREs core sequence as 5′-AUUUA-3′, often present in multiple copies [18]. The motif which was found most efficient in destabilizing mRNA was the nonamer UUAUUUA(U/A)(U/A) [19], although regions rich with A/U but with no core motif exist and can destabilize mRNA [20]. AREs are divided into three classes [16]. Class I contains several dispersed core motifs AUUUA in U-rich regions, class II contains sequences that have at least two overlapping nonamers UUAUUUA(U/A)(U/A) (this class can be divided into subgroups) class III contains U-rich regions with no defined AUUUA motif. It is important to note that no real consensus sequence, apart of the 5′-AUUU-3′ core, has been precisely defined for AREs. Because of this, ARE cannot be defined as PWMs, but still can be found using non-exact motif browsers. AREs classification was not based on biological functions (i.e. no association to proteins), but it is interesting that most of the mRNAs containing AREs class II encode cytokines, in contrast to mRNAs encoding transcription factors and cell cycle regulatory proteins which mostly contain AREs class I and sometimes class III. These differences in presence of different classes of AREs may suggest that they group together mRNAs from genes which can have different role, but are acting in similar regulatory pathways [20].

### Evolution of promoter regions and timing of activation of NF-κB-dependent genes

If the cofactor hypothesis concerning the timing of the response of NF-κB-dependent genes has some merit, as it is claimed in Paszek et al. [7], then it is likely that the differences in timing are reflected by differences in the structure of promoter regions of genes in different categories. Although Paszek et al. [7] work does not give detailed information on cofactors, we can depict three types of additional factors which may influence activation. One is DNA-binding specificity of combinations of NF-κB heterodimers [5], which allows two κB sites function together as a module, which differentially regulates gene activation. This phenomenon suggests that the configuration of half of binding sites may influence the effectiveness or the heterodimeric complex of NF-κB that it binds. Another factor is presence of the modifying transcription factors, such as AP-1 (or others), that amplify the effect of NF-κB binding. Still another is association of non DNA-binding proteins, such as p300-CBP coactivator family that are brought to the promoter by NF-κB, and act to increase expression of their target gene. p300-CBP increases expression by relaxing chromatin structure at the gene promoter through histone acetyltransferase (HAT) activity, recruiting RNA Polymerase II and other coactivators as adaptor molecules [21]. Specifically, these types of cofactors might discern differences in the number and type of transcription factor binding motifs required for NF-κB itself, as well as for the putative cofactors. This issue is best considered in the evolutionary framework; first, since functional binding sites are likely to be conserved in evolution, and second, since the patterns of evolutionary change of promoter regions are not very well-known and are of serious interest. Investigation of the structure and evolution of promoter regions of NF-κB dependent genes, in a set of related species, is the method we adopted to investigate and clarify the mechanism controlling dynamics of genes transcriptional response. This latter problem is the main topic of this paper.

Our paper expands the earlier study of Tian et al. [2] to include evolutionary comparisons of 4 species, human (Homo sapiens), chimpanzee (Pan troglodytes), mouse (Mus musculus) and cattle (Bos taurus). In this way we add to the most commonly used human-mouse comparison, two species, chimpanzee and cattle. See Discussion for more detail.

### Concepts of cis-regulation in eukaryotic genes

Cis-regulatory modules (CRMs) consist of clusters of TFBS which direct gene expression. They can be seen as a circuit relaying input signals into an output, which is gene activity, by combinatorial binding of TF. Although pattern discovery techniques allowed the effective identification of CRMs and corresponding TFBS for several single-celled organisms [22], applications in higher eukaryotes are not fully effective [23]. Flexible and combinatorial binding of TFBS can result in computational binding profiles of low specificity, which is the cause of difficulties with identification of regulatory regions embedded within long candidate regions [24]. Cross-species comparison of sequences from orthologous genes reduces the number of sequences under consideration and highlights conserved regions that are more likely to serve a biological function [25]. Based on cooperative work arising from clustering of TFBS, many computational methods have been created for identification of CRMs MOPAT, Cister, Cluster-Buster, CisPlusFinder, Crème, [26-31].

## Results

### Statistics of NF-κB-family binding motifs in NF-κB-dependent genes versus random sequences

To determine if there exists regulatory association between NF-κB family TFs and our dataset we used PASTAA software [32]. Results for top 10 associated TFs are presented in Table 1 and 2. As expected the highest affinity for gene set exists for NF-κB family members.

**Table 1:** Transcription factors association with gene data set

**Rank**	**Matrix**	**Transcription factor**	**Association score**	***p*-value**
1	NFKAPPAB65_01	Rela	15.083	0.00
2	NFKB_Q6_01	Nf-κb1, Nf-κb2	13.488	0.00
3	NFKAPPAB_01	Rela	13.336	0.00
4	CREL_01	C-rel	12.883	0.00
5	NFKB_Q6	N/A	11.200	0.00
6	NFKB_C	N/A	9.671	0.00
7	NFKAPPAB50_01	N/A	7.199	0.00
8	CDPCR1_01	Cutl1	5.367	8.30 × 10^-5^
9	CDPCR3HD_01	Cutl1	5.265	1.03 × 10^-4^
10	PAX2_01	Pax-2 , Pax-2a	4.845	2.66 × 10^-4^

PASTAA results for TFs associated with input set of genes ranked by *p*-values. Values were obtained for human sequences and human-mouse conserved sequence blocks. PASTAA settings: promoter sequence length closest to that considered in our study was 400 bp upstream TSS, affinity level: maximum affinity across promoter range.

**Table 2:** Transcription factors association with gene data set

**Rank**	**Matrix**	**Transcription factor**	**Combined**	**Corrected**
			***p*-value**	***p*-value**
1	NFKB_Q6_01	Nf-κb1 , Nf-κb2	6. × 10^-18^	3.57 × 10^-15^
2	NFKAPPAB65_01	Rela	7.31 × 10^-17^	2.02 × 10^-14^
3	CREL_01	C-rel	1.21 × 10^-16^	2.24 × 10^-14^
4	NFKAPPAB_01	Rela	1.68 × 10^-16^	2.32 × 10^-14^
5	NFKB_C	N/A	7.56 × 10^-14^	8.37 × 10^-12^
6	NFKB_Q6	N/A	1.44 × 10^-13^	1.33 × 10^-11^
7	NFKAPPAB50_01	N/A	5.08 × 10^-9^	4.02 × 10^-7^
8	WHN_B	Foxn1	2.15 × 10^-7^	1.49 × 10^-5^
9	DEAF1_01	Deaf-1	8.96 × 10^-6^	5.52 × 10^-4^
10	SP1_Q2_01	Sp1 , Sp2	1.61 × 10^-5^	8.91 × 10^-4^

TRAP results for TFs associated with input set of human promoter sequences. TRAP settings: background model: human promoters. *p*-Values for the individual sequences are combined using Fisher’s method. For multiple test correction Benjamini-Hochberg (BH) method was chosen. Matrix names correspond to the TRANSFAC database.

The basic descriptive statistics of the number of binding motifs found in the study, are collected in Table 3, which is listing group-by-group (rows) the number of motifs found (columns), for NF-κB-related genes itemized and for other sequences (random sequences and shuffled real promoter sequences) jointly. This study reveals that among chosen NF-κB-dependent genes, the average number of separated NF-κB-family TFBS detected in dataset equals to 6.07 per sequence, while the number in random sequences and shuffled sequences is about 2 TFBS. There is a considerably high percentage of NF-κB-related TFBS (multiple and overlapping) among the Early and Middle genes, in contrast to a lower number of NF-κB-related TFBS found in the promoters of Late genes. Wilcoxon test of abundance [33] (Table 4) shows that there is a statistically significant difference between the randomly generated and shuffled real promoter sequences and the promoter sequences of NF-κB-dependent genes in dataset. This comparison indicates that there exists a substantial difference between occurrence of NF-κB-related TFBS not only between NF-κB-dependent and random sequences, but also among the Early and Middle versus the Late groups.

**Table 3:** Participation of NF-κB-family binding motifs among human TFBS

**Group of genes**	**Number of TFBS found**				
	**NFκB**	**c-Re**l	**p50**	**p65**	
(A)					
Early	31	27	18	17	
Middle	32	34	16	20	
Late	18	22	14	12	
(B)					**Avg. number of TFBS**^**a)**^
Sum for dataset	81	83	48	49	**6.07**
Sum for 50 random sequences	28	49	15	26	**2.36**
Average sum for shuffled sequences	22.6	36.7	13	15.5	**2.09**

Numbers of separate TFBS found in the promoter regions of NF-κB-dependent genes (43 genes) and in randomly generated sequences (50 sequences). Part (A): counts of NF-κB family TFBS found in each group of genes in dataset. Part (B): Total counts of each type of TFBS in the dataset and number of motifs found in randomly generated sequences of length 1000 bp, and average sum of the motifs found in shuffled sequences (each sequence shuffled 10 times). Last column corresponds to the average number of NF-κB family TFBS found in one sequence. Motifs were found using NucleoSeq software which enables looking for distinct motifs; there is significant difference between number of TFBS found in real sequences and shuffled (*p* = 0.0286).

^a)^ Average number of TFBS per gene in dataset (Early, Middle or Late).

**Table 4:** ***p*-Values for abundance of NF-κB binding sites in gene data set versus random and shuffled sequences**

**Group of genes**	***p*-value**
Early	1.59 × 10^-5^
Middle	2.72 × 10^-6^
Late	6.36 × 10^-4^

Wilcoxon test for abundance of NF-κB binding sites in NF-κB related genes in comparison to shuffled and random sequences. The test shows that for the Early, Middle and Late genes there is a significant difference in the number of found NF-κB binding motifs.

### Systematic differences in promoter region structure in early versus middle versus late genes

Additional file 1: Table S1 is listing occurrence of different NF-κB-family members binding motifs found in promoter sequences. Occurrence of NF-κB-related human motifs is comparable to other species in all three groups of genes considered. Comparing the counts of motifs corresponding to any particular NF-κB-family TF, it is seen that NF-κB and REL TFBS are the most abundant in all types of genes and p65 is the least abundant one. For human and chimpanzee specific NF-κB binding motifs are more numerous than those of other NF-κB-family TFBS in the Early genes, but REL binding motifs are more abundant in Late and Middle genes. In case of more distant species, mouse and cattle, the situation is different. For mouse in all gene groups the REL-specific TFBS is the most abundant. For cattle gene groups, abundance of the REL-specific TFBS is higher in Early and Late genes, for the Middle genes NF-κB1-specific TFBS are most abundant. This confirms the expectation that NF-κB and REL play the most important role in regulation of expression of these genes.

Wilcoxon test shows that there is a significant difference between the number of NF-κB family TFBS found in the NF-κB-related compared to other sequences. In the Early genes group, in all the species, the number of NF-κB-family TFBS found is considerably higher than in the Late genes group; result for human promoters is presented in Table 5. In nearly all human and chimpanzee genes, this number exceeds 20 TFBS (counting overlapping motifs), with the exception of *EFNA1* with only 1 binding motif found, *CXCL2* with 6, *IL8* where the count of TFBS found is also low and equals 7 for both human and chimp and *CXCL3* with 15, whereas in the Late genes group 3 genes have more than 20 TFBS. In mouse and cattle genes similar pattern occurs. In the cattle Early genes group, the lowest number of NF-κB-family related TFBS was found for *IL8* with 8 motifs, and only 1 found for *EFNA1.* In mouse, which has no orthologue gene for *IL8*, lowest numbers of TFBS found are these for *EFNA1* (7) and *PLAU* (6).

**Table 5:** NF-κB - family TFBS found in promoter region of human genes

**Gene name**	**Distinct TFBS**	**All TFBS**	**TFBS index**^**a)**^
CCL20 (E)	6	22	**4.58**
CXCL1/Gro-a (E)	4	21	
CXCL2/Gro-b (E)	5	6	
CXCL3/Gro-g (E)	5	15	
EFNA1 (E)	1	1	
IL6 (E)	3	20	
IL8 (E)	1	71	
IRF1 (E)	8	50	
NFKBIA / IkBa (E)	7	44	
PLAU (E)	9	22	
PTGS2 (E)	5	24	
REL (E)	7	42	
TNF (E)	6	25	
TNFAIP3 (E)	6	35	
BCL3 (M)	5	30	**3.32**
BID (M)	5	6	
BIRC2 (M)	4	10	
CD83 (M)	7	19	
CFB / CompB (M)	7	29	
ECE1 (M)	7	14	
GCH1 (M)	2	4	
GFPT2 (M)	8	34	
IFNGR2 (M)	7	13	
KLRC3 (M)	0	0	
NFKB1/ NF-κB1 (M)	8	37	
NFKBIE / IkBe (M)	6	18	
RELB (M)	4	34	
SDC4 (M)	7	21	
SLC7A2 (M)	1	1	
SOD2 (M)	4	9	
TNFAIP2/ B94 (M)	4	9	
TRAF2 (M)	7	21	
ICAM1 (L)	5	13	**3.05**
IL27RA (L)	4	13	
IL32 / NK4 (L)	4	8	
NFKB2 (L)	11	29	
PTGES (L)	5	9	
TAP1 (L)	8	19	
TAPBP (L)	5	32	
TNIP1/ Naf-1 (L)	9	28	
TRAF1 (L)	0	0	
TRAF3 (L)	6	20	
TRIM16 (L)	4	15	

Results of search for NF-κB - family TFBS in human promoters in Early, Middle and Late genes. Gene name is presented in first column and affinity to the Early (E), Middle (M) and Late (L) genes group. Second column presents the number of distinct TFBS found across the promoter. Third column presents the number of all sites found using ConSite [13]. Last column presents ratio of average counts of all found motifs to average of distinct motifs per gene in given group of genes, which is called the motif overlap index

^a)^ TFBS motif overlap index

### Pattern of conservation and evolutionary change of TFBS in promoter regions in the context of species relatedness

Cross-species comparison revealed that conservation of NF-κB family - related TFBS motifs is much higher in the Early genes group than in the Late genes group. The highest numbers of common DNA binding motifs considered were found in the locations where the adjusted promoter sequences were highly conserved. For almost all Early genes, the NF-κB-family related TFBS motifs were conserved between most pairs of species, with the exception of comparison between mouse and cattle in TNF. As we presumed the best promoter sequence conservation and interspecies conservation of TF binding motifs persisted between human and chimp, followed in many cases by that between human and cattle. In the case of two Early genes, *REL* and *TNFAIP3* comparison, no conserved NF-κB-family related TFBS were found between chimpanzee or mouse and cattle. In human versus cattle comparison two single non-overlapping binding sites were found, but this is a low score in comparison with the number of conserved TFBS found in other Early genes. In human versus chimpanzee comparison in nearly all Early genes, all NF-κB-family related TFBS found were conserved. Only in the case of the *IκBα* gene the number of conserved TFBS is lower than the number of TFBS found separately for each of these species. The likely cause of this difference in promoter sequence is a long shift in promoter sequence alignment. Comparing given promoter sequences we can observe that in the case of the *IκBα* gene in human, the groups of TFBS found are located in the distant region of the promoter whereas in other studied species, NF-κB family-related TFBS are located in the proximal region of the promoter, and mostly conserved.

In the case of the Middle genes group, the highest number of conserved NF-κB-family related TFBS is found in the *RELB* gene. Among all species comparisons, conservation of this gene’s promoter sequences reaches 90% and more in the proximal region. Because of this, the most abundant multiple TFBS located close to the coding region are well conserved among all species. In the other Middle genes, *NFKB1* and *GFPT2* in which promoter structure is also similar to the early genes conservation of promoter sequences is weaker and accordingly the conserved NF-κB family-related TFBS are less numerous. Only between human and chimpanzee most of TFBS are conserved. Structure of TFBS looks similar when we analyse promoters separately, but in cross - species comparisons we discover lower sequence conservation, thus very low number or no conserved TFBS are found. In the *NFKB1* gene a similar arrangement of binding sites along the promoter sequence can be observed, and many NF-κB-family related TFBS are found in single promoter analysis, but cross-species comparison reveals quite low conservation of promoter sequences and visible differences between human and chimpanzee as compared to mouse and cattle promoter structures. No NF-κB family-related TFBS were found in human versus cattle and mouse versus cattle comparison due to very low overall promoter sequence conservation. However, chimpanzee versus cattle comparison revealed one common multiple NF-κB family-related TFBS. Similar results are observed for *GFPT2* gene, in which high conservation of TFBS is found between human and chimp, but in the other species promoter sequences are not well conserved and only two conserved TFBS have been found in human versus cattle and chimp versus cattle comparisons and one in mouse versus cattle comparison. The worst overall conservation in the Middle genes is observed in *TRAF2*, where even in the human versus chimpanzee comparison, one unique NF-κB-family related TFBS was found.

In the Late genes group cross-species comparisons, the lowest numbers of common conserved NF-κB family-related TFBS were found. This study revealed a great divergence between the promoter structures among considered species. Moreover there are only two genes, *NFKB2* and *TNIP1* in which the conserved NF-κB-family related TFBS can be found among all species. The best conservation results are between human and chimpanzee and between human and cattle. In other Late genes, usually only one or two cross-species comparisons reveal any existing common TFBS. Study of the Late genes group show that, if an orthologue gene exists, cattle promoter regions are filled with overall greater numbers of NF-κB family - related TFBS than in other species (*ICAM1, NFKB2, TRAF1, PTGES*). In human and chimpanzee *TRAF1* gene there is no NF-κB family-related TFBS and no conserved motifs were found between other species. In *TRAF3* there is a great similarity between human and mouse promoter, while a very low one between human and chimpanzee or human and cattle. Overall, the NF-κB family-related TFBS along the Late genes promoters sequences are distributed more sparsely than in the Early genes. TFBS are more scattered in promoter region and less of multiple and overlapping TFBS are found in comparison with the Early genes. The degree of sequence conservation between the Late genes in pairs of species also differs from that in Early genes, in most cases not exceeding 50%. We have also analysed the distribution of genes in view of the number of distinct and overlapping motifs and expression profile; results are presented in Additional file 1: Table S2.

**Figure 1:**
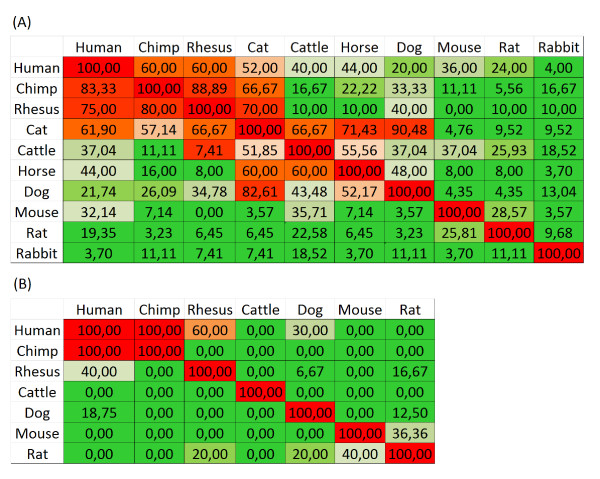
**Multiple cross-species comparisons for the representative of the Early gene TNF (A) and the Late gene ICAM1 (B).** The numbers in cells represent the percentage of NF-κB - family TFBS conserved between given species. Green colour depicts comparison with less than 50% conserved TFBS. In comparison (**B**), the number of species is only 7, since not for all species homologous gene was found. Column N, row M entry is the fraction TFBS conserved when promoter sequence of species N is aligned to the template being promoter sequence of species M. As a result the matrices in parts (**A**) and (**B**) are only approximately symmetric.

To check whether our choice of species covers a sufficient spectrum of evolutionary change, analysis involving additional 6 species has been carried out. For two sample genes from the Early and Late genes groups, promoter sequences of which were well-conserved in their groups, results are presented in Figure 1. Results suggest that 4 species: human, chimpanzee, cattle and mouse, chosen for analyses in this paper are sufficiently representative of evolutionary changes in promoter sequences of analysed NF-κB-dependent genes that evolved among wider groups of mammals. Indeed, comparison (A) in Figure 1 (Early gene) suggests that Human, Chimpanzee and Rhesus form a cluster with respect to the number of TFBS conserved; another such cluster is formed by Cat, Cattle, Horse and Dog, whereas Mouse, Rat and Rabbit differ markedly from each other. In comparison (B) (Late gene), the conservation structure is less tight.

We have also analysed the group of Biphasic genes which were presented in Tian et al. [2]; such group was not presented in Hao and Baltimore [34]. Six genes were analysed in the same way as the Early, Middle and Late genes in search for NF-κB family TFBS. We have observed that the Biphasic genes considerably differ with respect to the promoter structure and to the level of evolutionary conservation. Among the Biphasic genes there is one gene, *PSMB9*, in which we have found conserved TFBS in cross-species comparisons of all species considered. In *CYB5A* and *IFI35* human and chimpanzee have high number of overlapping and distinct TFBS, similarly as in the Early genes, while cattle and mouse have considerably less TFBS for the NF-κB family, which is more typical of the Late genes. For the *MVP* gene we have found only one TFBS in the human promoter, none in the mouse, but a high number of overlapping sites have been found in cattle. Overall conservation of promoter sequences in the Biphasic genes is low, usually not exceeding 50%, which is also the case for the Late genes. Only human/chimpanzee comparisons show very high conservation of sequences, which implies conservation of nearly all TFBS for NF-κB family. An exception is the *MVP* gene, for which chimpanzee does not have a homologous gene, and *PSMB8* for which the aligned sequence is short and does not contain any common TFBS. Results of the cross-species comparisons can be found in Additional file 1: Table S3.

### Analysis of 3′ UTR fragments with respect to ARE contents

**Figure 2:**
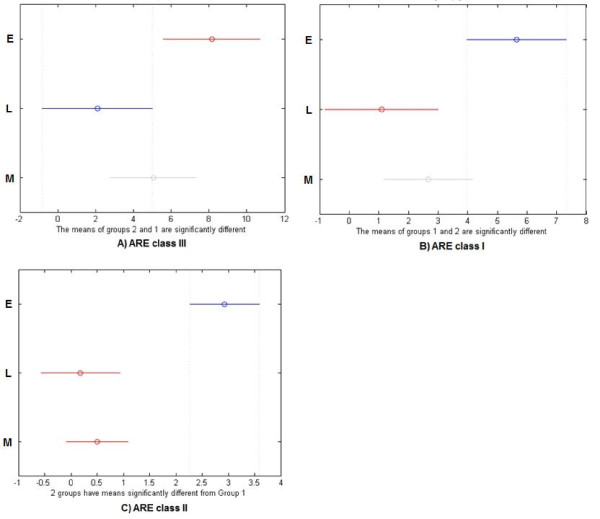
**Statistical comparison of numbers of AREs among the different classes of genes.** ANOVA test was applied to determine differences between occurrences of AREs in 3′UTRs of different classes’ genes: Early (E), Late (L) and Middle (M). Charts correspond to the type of ARE, **A:** class III, **B:** class I and **C:** class II. Early group genes always have significantly higher count of AREs than Late genes, and with growing complexity of AREs, Early genes also become more distant from Middle genes. Horizontal axis: count of ARE motifs found in gene groups. The circles denote mean value, the horizontal lines denote the confidence interval for mean value.

**Figure 3:**
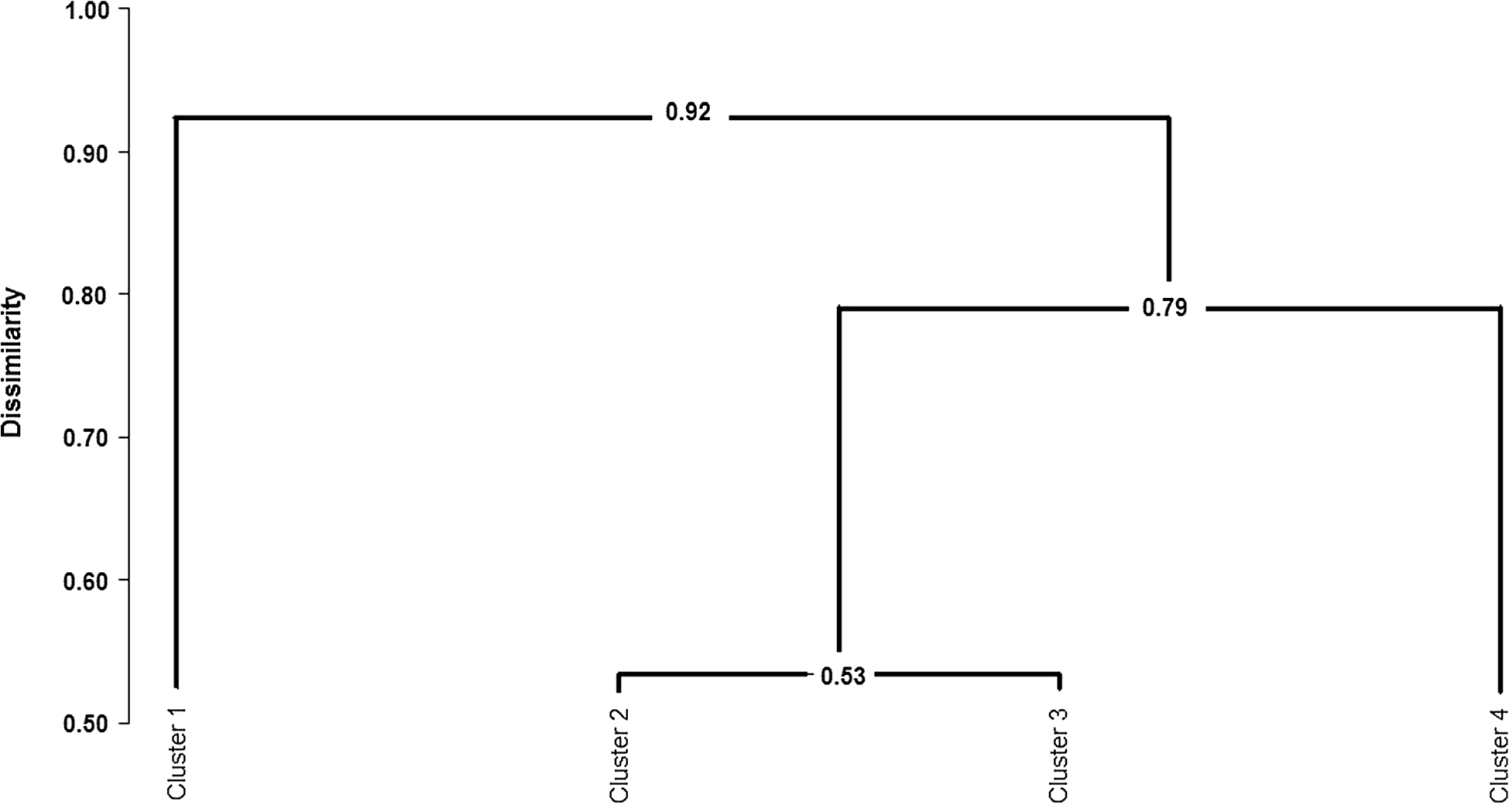
**Distribution of ARE in Biphasic genes.** Numbers of ARE motifs of each class for all genes from the biphasic group in human, cattle and mouse. ARE class III – no specific motif, ARE I – ‘ATTTA’ and repetitions of this motif, ARE II – repetitions of the nonamer TTATTTA(T/A)(T/A).

Taking into account results from Hao and Baltimore work, 3′ UTR fragments from the set of genes in by Tian et al. [2] were analysed. Computational results of this analysis agree with those presented by Hao and Baltimore [34]. Genes classified by Tian et al. as the Early genes have generally a larger number of ARE of each type in 3′ UTRs than the Late genes group, the difference is statistically significant at α = 0.05, with *p* = 1.43 × 10^-5^ for ARE class II, *p* = 1.02 × 10^-2^ for ARE class I, *p* = 3.42 × 10^-2^ for ARE class III (Figure 2). Depending on the class of ARE, which corresponds to complexity of motif, genes belonging to the Middle group are more or less similar to the Late group. The relation noted by Hao and Baltimore [34], connecting number of ARE, which destabilize mRNA, with regulation of gene expression, was tested computationally in other species considered in this study. In all four species early genes have significantly higher number of ARE elements. Analysis of 3′UTR was conducted by counting and comparing number of ARE’s motifs corresponding to different classes of AREs, without alignment of 3′UTR sequences, results for representatives of the Early, Middle and Late genes are presented in Table 6 (complete results are presented in Additional file 1: Table S4). NF-κB dependent genes were divided according to given factors. In Human, with few exceptions (*EFNA1, REL*) Early genes are a homogenous group with rich promoter and higher number of all types of AREs, especially considering more complex ARE motifs (class I, II). Late genes tends to have lower number of class III AREs and almost no class II AREs, only *TAPBP* and *NFKB2* have features similar to those in Early genes. Finding obvious correlation between number of NF-κB family binding sites and abundance of AREs is difficult, but clustering analysis based on expression pattern, number and scattering of binding sites and number of all three classes of AREs shows that Middle genes are divided in two groups, while Early and Late genes are separated (Figure 3, Table 7).

**Table 6:** Are motifs in the early, middle and late genes

**Human**	**ARE III**	**ARE II**	**ARE I**	**Chimpanzee**	**ARE III**	**ARE II**	**ARE I**
TAPBP (L)	8	0	3	TAPBP (L)	7	0	3
TRAF1 (L)	1	0	1	TRAF1 (L)	2	0	1
TRAF3 (L)	2	0	3	TRAF3 (L)	1	0	1
PTGES (L)	1	0	1	PTGES (L)	1	0	1
ICAM1 (L)	1	0	2	ICAM1 (L)	0	0	0
IL27RA (L)	3	0	0	IL27RA (L)	0	0	0
NFKB2 (L)	1	0	1	NFKB2 (L)	-	-	-
BID (M)	6	0	2	BID (M)	12	0	6
BIRC2 (M)	5	0	3	BIRC2 (M)	4	0	3
CFB (M)	1	0	0	CFB (M)	0	0	0
NFKBIE (M)	0	0	0	NFKBIE (M)	0	0	0
TRAF2 (M)	1	0	0	TRAF2 (M)	-	-	-
SOD2 (M)	2	0	2	SOD2 (M)	1	0	0
GCH1 (M)	13	0	11	GCH1 (M)	6	0	6
NFKB1 (M)	7	0	3	NFKB1 (M)	7	0	3
SDC4 (M)	10	0	3	SDC4 (M)	9	0	3
SLC7A2 (M)	24	1	12	SLC7A2 (M)	0	0	1
TNFAIP3 (E)	14	1	5	TNFAIP3 (E)	13	1	5
PTGS2 (E)	27	5	22	PTGS2 (E)	25	5	22
IL6 (E)	8	3	6	IL6 (E)	8	3	6
IL8 (E)	16	2	9	IL8 (E)	15	1	9
TNF (E)	2	5	9	TNF (E)	0	0	0
CXCL1 (E)	6	1	5	CXCL1 (E)	6	1	6
CXCL2 (E)	5	4	10	CXCL2 (E)	5	4	11
CCL20 (E)	5	0	3	CCL20 (E)	5	0	3
**Mouse**	**ARE III**	**ARE II**	**ARE I**	**Cattle**	**ARE III**	**ARE II**	**ARE I**
TAPBP (L)	3	0	0	TAPBP (L)	3	0	4
TRAF1 (L)	0	0	1	TRAF1 (L)	0	0	0
TRAF3 (L)	5	0	3	TRAF3 (L)	1	0	2
PTGES (L)	3	0	0	PTGES (L)	1	0	0
ICAM1 (L)	1	0	4	ICAM1 (L)	1	0	2
IL27RA (L)	2	0	0	IL27RA (L)	2	0	0
NFKB2 (L)	0	0	1	NFKB2 (L)	1	0	1
BID (M)	2	0	0	BID (M)	1	0	0
BIRC2 (M)	5	0	1	BIRC2 (M)	-	-	-
CFB (M)	1	0	0	CFB (M)	1	0	0
NFKBIE (M)	1	0	0	NFKBIE (M)	2	0	0
TRAF2 (M)	0	0	1	TRAF2 (M)	1	0	0
SOD2 (M)	-	-	-	SOD2 (M)	5	1	2
GCH1 (M)	5	1	7	GCH1 (M)	5	1	4
NFKB1 (M)	9	0	4	NFKB1 (M)	6	0	2
SDC4 (M)	6	0	1	SDC4 (M)	8	0	4
SLC7A2 (M)	17	1	12	SLC7A2 (M)	0	0	0
TNFAIP3 (E)	6	1	5	TNFAIP3 (E)	0	0	0
PTGS2 (E)	16	4	12	PTGS2 (E)	9	4	11
IL6 (E)	7	1	5	IL6 (E)	9	2	6
IL8 (E)	-	-	-	IL8 (E)	13	4	11
TNF (E)	3	5	8	TNF (E)	4	5	10
CXCL1 (E)	7	0	5	CXCL1 (E)	-	-	-
CXCL2 (E)	8	0	7	CXCL2 (E)	-	-	-
CCL20 (E)	3	0	5	CCL20 (E)	0	0	0

Counts of ARE class I, II and III in human, chimpanzee, cattle and mouse. Genes presented in tables are representatives of the Late (L), Middle (M) and Early (E) group. Columns 2–4 correspond to the type of ARE .Corresponding charts are presented in Additional file 2: Figures S5, Additional file 3: Figure S6, Additional file 4: Figure S7. Complete results are presented in Additional file 1: Table S4.

**Table 7:** Clustering of genes based on gene expression pattern, promoter structure and 3′UTR structure

**Cluster**	**1**	**2**	**3**	**4**
**Size**	**14**	**10**	**8**	**11**
	CCL20 (E)	BIRC2 (M)	BCL3 (M)	ICAM1 (L)
	CXCL1 (E)	CD83(M)	BID (M)	IL27RA (L)
	CXCL2 (E)	ECE1 (M)	CFB (M)	il32 (L)
	CXCL3 (E)	GCH1 (M)	KLRC3 (M)	NFKB2 (L)
	EFNA1 (E)	GFPT2 (M)	NFKBIE (M)	PTGES (L)
	IL6 (E)	IFNGR2 (M)	RELB (M)	TAP1 (L)
	IL8 (E)	NFKB1 (M)	TNFAIP2 (M)	TAPBP (L)
	IRF1 (E)	SDC4 (M)	TRAF2 (M)	TNIP1 (L)
	NFKBIA (E)	SLC7A2 (M)		TRAF1 (L)
	PLAU (E)	SOD2 (M)		TRAF3 (L)
	PTGS2 (E)			TRIM16 (L)
	REL (E)			
	TNF (E)			
	TNFAIP3 (E)			

Result of clustering based on three main features of data set, including gene name and group to which it was assigned in Tian et al. [2] experiment.

**Figure 4:**
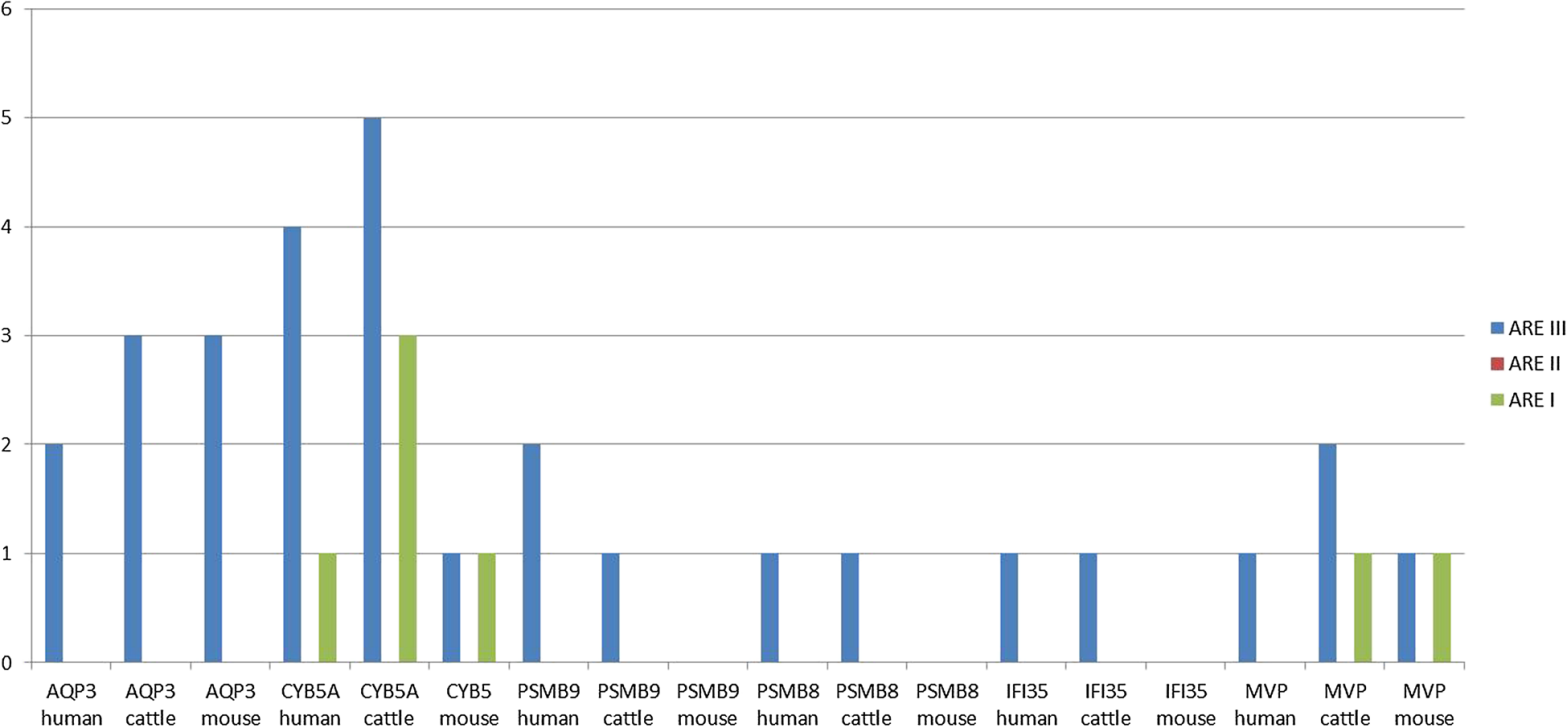
**Clustering of genes based on: gene expression pattern, promoter structure and 3′UTR structure.** Promoter structure: clustering acquired based on dispersion and richness (multiple versus overlapping) of NF-κB-family binding motifs in the promoter region and on occurrence of AREs in 3′UTR. Cluster 1 contains all Early genes, clusters 2 and 3: Middle genes, cluster 4: Late genes. Clustering method: percent disagreement, aggregation criterion: unweighted pair-group average.

Analysis of the Biphasic genes shows that this group’s members have even less ARE elements in the 3′ UTR than the Late genes. Results of the 3′UTR analysis are shown in Figure 4. ARE class II motifs are not present in the Biphasic genes, similarly as in the Late genes, but neither the ARE class I motifs have been found in the Biphasic genes, except for *CYB5A* and *MVP*. In *CYB5A*, 3 ARE class I motifs were found in cattle and 1 in the remaining species; in *MVP* gene only 1 was found in cattle and mouse. ARE class III are also scarce with the highest number of 5 found in cattle *CYB5A*.

## Discussion

### Structure, function and evolution of promoter regions in view of data concerning NF-κB-dependent genes

Comparison of the Early, Middle and Late genes groups reveals that strongest similarities among species can be found in the Early genes promoters. The Early genes group has the highest conservation percentage of promoter sequences and good sequence alignments, which is the cause of very good cross-species conservation of NF-κB-family related TF binding motifs. During evolution these non-coding sequences have maintained a very similar structure, which can serve as a proof of important regulatory functions [35] of expression of these genes that have not changed significantly over tens of millions of years since the divergence from a common ancestor. Moreover, this may suggest that the regulation pattern of these group of genes may have an effect on the result of gene expression and may be more universal, therefore likely to be shared between other species not included in this study.

Analyses of the Late genes expose significant differences in the promoter structure, number and location of NF-κB-family related TFBS in promoter sequence and a low number, if any, of conserved NF-κB-family related TFBS. This suggests that during evolution, promoter sequences of Late genes became more species-specific and the way that regulation of gene expression is accomplished has been relatively quickly changing with increasing evolutionary distance. Comparing human and chimpanzee promoters sequences with those of cattle and mouse, suggests that in the case of Late genes some NF-κB-family related TFBS lost their functionality and were abandoned or reorganized during species evolution.

**Figure 5:**
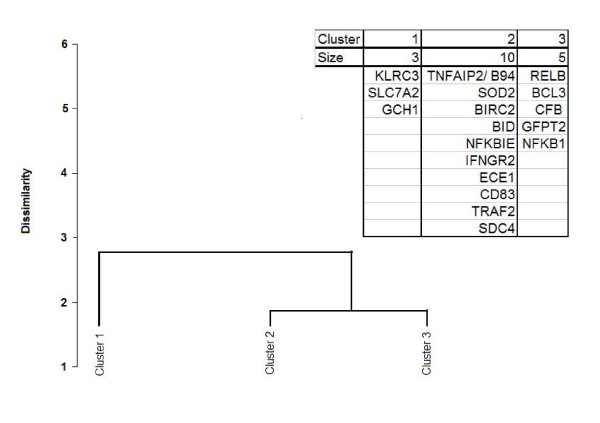
**Distribution of human Middle genes.** Result of agglomerative hierarchical clustering, method: Euclidean distance, agglomeration: unweighted pair-group average. Tabular inset shows genes affiliations to clusters. Cluster 1: very low number of TFBS, cluster 2: average number of TFBS, cluster 3: multiple and overlapping TFBS.

We noted that the Middle genes can be split into two groups: 1. Early-like, the promoter regions of which contain a relatively high number of clustered NF-κB-family TFBS similarly as in the Early genes, and 2. the Late-like genes, which contain a low number of not significantly clustered NF-κB-family TFBS in their promoters similarly as in the Late genes. To be exact, inspection of the hierarchical clustering patterns shows three groups, with two groups more related to each other than to the third one (as depicted in Figure 5, which also shows that Middle gene promoter regions are relatively rich in NF-κB-family binding motifs compared to Early genes).

For the Biphasic genes, results of computational analyses were unclear, because of a small number of genes in this group and considerable differences within this group with respect to sequence conservation level, number of TFBS in the promoter region and of the ARE motifs in the 3′UTR. By using computational methods only we were not able to find distinctive signatures, which might explain the dynamics of transcriptional response. The overall scarcity of the ARE elements is consistent with the Biphasic genes’ protracted expression, but the significance of this association seems unclear.

In non-NF-κB-dependent genes, NF-κB-family related TFBS generally represented less than 2% of all TFBS and were single, non-clustered sites. It has been suggested by Wunderlich and Mirny [15] based on information-theory considerations that sites of such structure are non-functional, since non-clustered binding sites may not be recognizable by the corresponding TFs. In the NF-κB-dependent genes and particularly in the Early and the Middle groups, the NF-κB family-related TFBS are much more numerous (around 10%) and are usually clustered together.

### Timing of gene activation

Our study revealed that promoter structures in the Early, Middle and Late genes are differently conserved in four species. The Early genes were found to have more NF-κB-family related TFBS, among which most are multiple and located close to each other, compared to genes belonging to the Late group. The cofactor hypothesis [7] may not be a sufficient explanation of such difference in expression timing between the Early, Middle and Late genes. Since eukaryotic TFs have low specificity, and are not as precisely targeted to functional cognate sites as prokaryotic TFs are [15], there is a possibility that, shortly after NF-κB-family related TFs are released in the nucleus, they are unable to locate functional binding sites in genes classified as Late. Because of that low specificity NF-κB family-related TFs may bind to non-functional sites, delaying the time of gene expression. Without a specific signal to express the gene, further TF molecules may continue to bind to the available and recognizable binding motifs. Somewhat similarly as in Paszek et al. [7] cofactor hypothesis, the Late genes may require more than one functional binding site to be bound by TF to start expression, and the higher chance of TFs binding to non-functional sites is the reason that a longer time is required before TF reaches the functional cognate sites.

In the Early genes among all four species, NF-κB family-related TFBS are multiple and closely located. This may significantly improve functional binding site recognition for TF, resulting in much faster binding to the accurate cognate spot than it occurs in the Late genes. Proximity of multiple, less specific TFBS in Early genes may be a cause of synergistic transactivation of NF-κB dependent genes, and therefore one of the causes of faster gene activation. It may be strongly connected with creating κB binding modules. Synergistic binding was proven for glucocorticoid receptor in Wright and Gustafsson work [36] and studies in Ron et al. [37] have suggested that there are interactions between NF-κB and other adjacent regulatory factors, so other proteins may bind the same site as does NF-κB. The genes referred to as ‘Early’, because of multitude of functional binding sites, are less ‘TF-specific’ and may allow more than one combination of recognized TF to activate gene, which may accelerate gene expression.

According to Hao and Baltimore [34] the stability of mRNA influences the timing of gene activation. Results presented in their paper are similar to those obtained by Tian et al. [2]. They categorized TNF-activated genes into three major groups which correspond to the Early, Middle and Late genes of Tian et al. [2]. Although a slight shift in peak of the expression timing is visible in comparison to Tian’s research, group I (corresponding to the Early group) genes peaked at approximately 30 min, group II (corresponding to the Middle group) peaked at 2 hrs. and group III (corresponding to the Late group) peaked at 12 hrs. The expression of the groups was overall qualitatively similar. This similarity in these two experiments led us to seek a correlation between promoter structure described above, and the occurrence of ARE elements in the 3 ′UTR sequence. Despite the fact that sets of genes in the two experiments cited [2,34] are not identical, 14 genes can be found in both studies. Eight of these 14 genes have been assigned to the Early group in both studies compared to the only two representatives of the Late group (Table 8). The analysis of the 3′ UTR fragments in Tian et al. [2] genes shows that the presence of ARE in the 3′UTR of the analysed genes also acts as a regulatory effect on the gene activation timing. However it is not known which element if any, the structure of the promoter or the 3′UTR has a dominant role in that process or if they are equally co-responsible and more research should be conducted to explain this problem.

**Table 8:** Classification of genes common to Tian et al. and Hao & Baltimore studies

***Gene name***	***Classification based on ARE count (mRNA stability) ***[34]	***Classification based on expression pattern ***[2]
PTGS2	Early	Early
CXCL1	Early	Early
CXCL2	Early	Early
IL6	Early	Early
TNFAIP3	Early	Early
NFKBIA	Early	Early
IRF1	Early	Early
**ICAM1**	**Middle/ Early**	**Late**
**CCL20**	**Middle/Early**	**Early**
IFNGR2	Middle	Middle
NFKBIE	Middle	Middle
RELB	Middle	Middle
TNFAIP2	Middle	Middle
TAPBP	Late	Late

Classification of genes common to Hao and Tian studies. Columns: classification based on ARE occurrence in 3′UTR [34], and classification based on expression timing after TNF simulation [2]. Borderline genes shown in bold.

## Conclusions

This study is a follow-up to Tian et al. [2] work, which provided us with the list of NF-κB-dependent genes; in addition we compare Tian et al. [2] results with Hao and Baltimore work [34] on mRNA stability determining different transcription kinetic patterns. In this study we conducted phylogenetic analysis, not only for human and mouse as in ref. [2], but also for chimpanzee and cattle. This design allows a better insight into the changes that occurred during evolution. Cross - species similarities in promoter regions of chosen genes, which are depicted by corresponding TFBS for NF-κB family transcription factors in orthologous genes, are a part of this study. Using this approach we analysed if gene’s assignment to the Early, Middle or Late group based on expression pattern is connected with special features in promoter structure. In this study not only specific NF-κB binding sites were considered but also the binding sites for p50, p65 and cRel, which show clustering of TFBS among Early genes. Wider phylogenetic analysis has shown that Early genes of even relatively distant species, such as human and cattle, share many similarities in promoter structure. This indicates important evolutionary conservation of the regulatory role of these genes. We also analysed the promoter regions of Late genes in all species considered and found that promoter structures were different from those in Early genes, even between closely related species such as human and chimp. This may indicate different regulatory patterns for the Late genes. In a recent publication [15] it was demonstrated computationally that TFBS clustering increases efficiency of transcription activation. This is consistent with TFBS clustering observed by us in Early genes. Our findings were compared to recent information on TF binding [15,38], and this suggests that Early gene expression may be the result of TFBS clustering which results in faster recruitment of NF-κB and, conversely, it is a feature that may help distinguish functional binding sites from spurious ones. Similarly, in a recent paper [39] it was shown that evolution might favor multiple TFBS even if the affinity to the TF molecule is weaker. Analysis of 3′UTRs in our gene set also shows that genes assigned to Early, Middle and Late group differ significantly with respect to number of AREs found, with Middle genes group being divided into Early-like and Late-like subgroups, but not having both features at once. This connection may be one of the mechanisms underlying the different patterns of gene expression control; it was already briefly discussed in Tian et al. [2]. Although the ARE consensus motif is not completely defined we analysed 3′UTRs with motifs corresponding to each class which allowed us to perform wide search of AREs in all genes. We observed that for any given motif type, the highest count of AREs was found in the Early group genes opposed to Late group genes, where only simple AREs class III and in few cases AREs class I were found in relatively larger number. As gene data sets considered in Tian et al. [2] and Hao and Baltimore [34] are different, in the future a phylogenetic study can be extended to genes presented by Hao and Baltimore [34], to examine the promoter structures. Experiments combining different types of promoters with 3′UTR containing different number of destabilizing ARE units might be carried out, in order to check if putting together elements connected with different expression patterns could alter expression timing. Extending the gene dataset to more NF-κB-dependent and interferon dependent genes, will provide knowledge about gene expression mechanisms and control innate immunity.

Summing up, our results suggest that the correlation between promoter region structure and ARE occurrence in 3′UTR, contributes important knowledge about gene expression patterns. We can hypothesize that in the Early genes, early initiation of transcription (determined largely by dense promoter TFBS packing) is followed by quick termination (determined by abundance of ARE elements in the 3′UTR). The opposite is true in the Late genes where late initiation of transcription (determined largely by loose promoter TFBS packing) is followed by slow termination (determined by scarcity of ARE elements in the 3′UTR). If confirmed, this constitutes an interesting adaptation, playing a role in temporal control of transcription.

## Methods

### Bioinformatic tools for motifs discovery

There are a few available web-based applications such as ConSite [13], rVista [40], oPOSSUM [41], FootPrinter [42] or PASTAA [32] which enable phylogenetic footprinting and TFBS detection. As we needed to make interspecies comparison we decided to use ConSite and rVista, which allows for unrestricted sequence comparison. We also used PASTAA to check TFs affinity for our input gene set. For a thorough analysis of promoter sequences we selected ConSite as the most flexible for our needs and applied it to search for the NF-κB family TFBS in retrieved promoter regions of the genes selected and to detect conserved motifs shared between human sequences and their orthologs in chimpanzee, mouse and cattle genomes. Binding sites hits were re-checked using rVista. Revision enabled better recognition of non - overlapping sites, but many indications for overlapping sites were lost. This is due to rVista having stronger constraints set for searching for TFBS in promoter sequence. As the interactive expert system for retrieving orthologue sequences in ConSite was temporarily disabled, all sequences were recovered from the UCSC Genome browser. The first step was the search for NF-κB-related TFBS in promoter sequences of each gene, to determine the base number of the TFBS found in that region, for all species. The second step was the cross-species comparison of orthologous promoter sequences to determine the number of conserved NF-κB-family TFBS. ConSite and rVista use matrix threshold to match motifs to PWM (position weight matrix). Sites are scanned by sliding the corresponding PWM along the sequence and scoring it at each position. The threshold is the minimum relative score used to report the position as a putative binding site. In all cases, PWM threshold was set to 80% to avoid finding weak TF binding motifs; for pair-wise comparisons sliding window length was 50 nucleotides, whereas the conservation cut-off was set to 40% to assure sufficient length of sequences to be searched.

ARE analysis was conducted using NucleoSeq [43] which is a user-friendly application that allows downloading, storage, and analysis of the sequences of mRNA transcripts. Sequences were automatically downloaded from the RefSeq database based on EntrezGene ID or gene symbol. Several ARE motifs were used representing class I, II, III and core motif presented by Hao (ATTT).

### Databases employed

UCSC Genome Browser was used to retrieve human 1000 bp 5′ sequences to the first exon, which we call here promoter sequences. Promoter regions for other analysed species were retrieved by BLATing human sequence to other species genome and by analysing synteny blocks. If no significant match was found, then sequence 1000 bp upstream from TSS was assumed to represent promoter region for a certain gene. Human (Homo sapiens) and chimpanzee (Pan troglodytes) genes were recovered from the March 2006 genome assembly, mouse (Mus musculus) genes from the July 2007 assembly, and cattle (Bos taurus) genes from the October 2007 assembly. Ensembl database was employed for acquisition of some of chimpanzee and cattle genes. Profiles of chosen TFs were drawn from the JASPAR database and then converted to log-scaled position weight matrices (PWMs) in order to evaluate possible binding sites in the input sequence [13].

### Selection of animal species

For this research four mammalian species were chosen with their evolutionary distance from humans as the main guideline. Chimpanzee was chosen as the closest to human (common ancestor about 5 million years ago), to inspect hypothetically most recent changes in TFBS conservation. On the other hand, mouse genome is close to human whereas the evolutionary distance is much larger about 75 million years to the common ancestor; [44], which provides a better insight into conservation patterns. Cattle was chosen as the most distant from human (about 90 million years) in this comparison; however it still maintains many similarities to the human sequences, i.e. protein sequences that are closer than those found in mice and “because the cattle diverged from the human branch so long ago, analysis of its genome makes it possible to identify which human traits are well-conserved.” [45].

### Selection of genes: early, middle and late genes

In this work, promoter regions of NF-κB dependent genes 1000 bp in length were used to establish the conservation profile of the NF-κB family TFs among humans and three mammalian species. From the set of 74 uniquely NF-κB-dependent genes [2], we have chosen groups of the Early (14), Middle (18) and Late (11) genes. In comparison to Tian et al. [2] we excluded one gene assigned to the Late genes, *KLRC2*; according to the HomoloGene database there is no homologue genes for species considered in our paper. Genes in dataset are assigned to groups according to Table 3 in Tian et al. [2]. The genes selected represent a spectrum of principal molecular functions of NF-κB-dependent genes such as cytokine activity in Early genes or protein binding and signalling in Late and Middle genes. The set of analysed genes is also connected to the NF-κB signalling pathway. The list of genes is found in Table 3.

### Selection of binding motifs

All retrieved gene promoter sequences were searched, using ConSite and rVista software, for occurrence of four mammalian NF-κB related binding motifs: NF-κB (heterodimer p50/p65), REL (cREL), p50 (NF-κB1), p65 (RELA). TF detection threshold set for 80% is a value the purpose of which is the optimal compromise between the sensitivity of motif detection and its specificity. As hit-based prediction is not the only method for finding TFBS, to strenghten our findings we employed PASTAA and TRAP web tools to determine affinity for this transcription factors in our gene set. PASTAA ranks genes based on the annotated binding affinities of TRANSFAC TFs database to genes promoter regions. It also ranks genes based on their tissue specificities derived from expression data. PASTAA retrieves promoter sequence based on gene ID, TRAP works with given sequence dataset. Then in each single-gene promoter analysis and in cross-species comparisons, the numbers of detected TFBS were summarized and then compared to each other respectively. This strategy allowed us to distinguish how many common TFBS are conserved and where they are localized in the promoter sequence.

To determine the importance of NF-κB-related binding motifs on the background of all human-specific TFBS, found by ConSite, in a given NF-κB-dependent gene dataset, we carried out a comparative study. We determined the occurrence of NF-κB-related binding motifs in a set of 9 randomly chosen non-NF-κB dependent genes. We also searched 50 random sequences generated by NucleoSeq for NF-κB family TFs. The rationale for the study is that if the NF-κB-related binding motifs found by us were mostly non-functional, then there would be likely no difference of their occurrence between the NF-κB-dependent and non-NF-κB-dependent genes.

## Competing interest

The authors declare that they have no competing interests.

## Authors’ contributions

MI designed and carried out the analyses and wrote the paper. ARB contributed to the design of the analyses and verified the biological set-up. MK conceived the study and contributed to the design of analyses. All authors read and approved the final manuscript.

## Supplementary Material

Additional file 1 **Supplemental Tables.** File contains set of supplemental tables. Click here for file

Additional file 2 Figure 5. Click here for file

Additional file 3 Figure 6. Click here for file

Additional file 4 Figure 7. Click here for file
