# Supplementary material for: The dependence of expression of NF-κB-dependent genes: statistics and evolutionary conservation of control sequences in the promoter and in the 3′ UTR
Source: BMC Genomics. 2012 May 11;13:182. doi: 10.1186/1471-2164-13-182 (PMC3488004; doi:10.1186/1471-2164-13-182)
Supplement: Additional file 1 — Supplemental Tables. File contains set of supplemental tables. [file 1471-2164-13-182-S1.doc]

| **The dependence of expression of NF-κB-dependent genes: Statistics and evolutionary conservation of control sequences in the promoter and in the 3’ UTR** |
| --- |
| Supplemental Tables |
|  |
| **Marta Iwanaszko, Allan R. Brasier, Marek Kimmel** |

|  |
| --- |

## Supplemental Table 1 - Summary of counts of NF-κB - family related motifs

Counts of NF-κB-family related motifs found in the Early, Middle and Late genes for the four species. Numbers relate to overlapping motifs. Underlined number is the most numerous TFBS found in a given group of genes.

| a) Human |  | | | |  | |  | | |  | |  |
| --- | --- | --- | --- | --- | --- | --- | --- | --- | --- | --- | --- | --- |
|  | **NFkappaB** | | | | **cRel** | | **p50** | | | **p65** | |  |
| **Total count of NFkB-family related motifs** | 258 | | | | **263** | | 166 | | | 140 | |  |
|  |  | | | |  | |  | | |  | |  |
| Early genes | **104** | | | | 101 | | 77 | | | 52 | |  |
|  |  | | | |  | |  | | |  | |  |
| Middle genes | 93 | | | | **100** | | 59 | | | 57 | |  |
|  |  | | | |  | |  | | |  | |  |
| Late genes | 61 | | | | **62** | | 32 | | | 31 | |  |
| b) Chimpanzee | |  | | |  | |  | | |  | | |
|  | | **NFkappaB** | | | **cRel** | | **p50** | | | **p65** | | |
| **Total count of NFkB-family related motifs** | | 270 | | | **286** | | 206 | | | 152 | | |
|  | |  | | |  | |  | | |  | | |
| Early genes | | **104** | | | 101 | | 81 | | | 56 | | |
|  | |  | | |  | |  | | |  | | |
| Middle genes | | 112 | | | **118** | | 88 | | | 64 | | |
|  | |  | | |  | |  | | |  | | |
| Late genes | | 54 | | | **67** | | 37 | | | 32 | | |
| c) Mouse | | |  | |  | |  | |  | | |  |
|  | | | **NFkappaB** | | **cRel** | | **p50** | | **p65** | | |  |
| **Total count of NFkB-family related motifs** | | | 252 | | **263** | | 166 | | 159 | | |  |
|  | | |  | |  | |  | |  | | |  |
| Early genes | | | 101 | | **109** | | 63 | | 68 | | |  |
|  | | |  | |  | |  | |  | | |  |
| Middle genes | | | **90** | | **90** | | 65 | | 53 | | |  |
|  | | |  | |  | |  | |  | | |  |
| Late genes | | | 61 | | **64** | | 38 | | 38 | | |  |
| d) Cattle | |  | |  | |  | |  | | |  | |
|  | | **NFkappaB** | | **cRel** | | **p50** | | **p65** | | |  | |
| **Total count of NFkB-family related motifs** | | 267 | | **279** | | 154 | | 154 | | |  | |
|  | |  | |  | |  | |  | | |  | |
| Early genes | | 102 | | **105** | | 51 | | 58 | | |  | |
|  | |  | |  | |  | |  | | |  | |
| Middle genes | | **101** | | 97 | | 72 | | 58 | | |  | |
|  | |  | |  | |  | |  | | |  | |
| Late genes | | 64 | | **77** | | 31 | | 38 | | |  | |

## Supplemental Table 2 - Hierarchical distribution of genes based on number and type of TFBS in promoter region

Table shows distribution of genes after agglomerative hierarchical clustering for NF-κB dependent genes. Clustering is based on the number of separated TFBS, overall count of TFBS (multiple overlapping) and assignment to the Early, Middle or Late genes in the experiment. Method: Euclidean distance, unweighted pair-group average aggregation. Data have been standardized. Cluster 1 contains genes with no or a very low number of TFBS. Cluster 2 contains genes assigned to the Late group which have rich promoter regions. Cluster 3 contains genes with high numbers of TFBS distributed in the promoter region (most of them assigned to the Early genes). Cluster 4 contains genes with medium number of TFBS (it includes Late and Middle genes).

| **Cluster** | **1** | **2** | **3** | **4** |
| --- | --- | --- | --- | --- |
| **Size** | **6** | **2** | **17** | **18** |
|  | EFNA1 | NFKB2 | CCL20 | BID |
|  | IL8 | TNIP1 | CXCL1/Gro-a | BIRC2 |
|  | GCH1 |  | CXCL2/Gro-b | CD83 |
|  | KLRC3 |  | CXCL3/Gro-g | ECE1 |
|  | SLC7A2 |  | IL6 | IFNGR2 |
|  | TRAF1 |  | IRF1 | NFKBIE |
|  |  |  | NFKBIA | SDC4 |
|  |  |  | PLAU | SOD2 |
|  |  |  | PTGS2 | TNFAIP2 |
|  |  |  | REL | TRAF2 |
|  |  |  | TNF | ICAM1 |
|  |  |  | TNFAIP3 | IL27RA |
|  |  |  | BCL3 | Il32 |
|  |  |  | CFB | PTGES |
|  |  |  | GFPT2 | TAP1 |
|  |  |  | NFKB1 | TAPBP |
|  |  |  | RELB | TRAF3 |
|  |  |  |  | TRIM16 |

## Supplemental Table 3 - Conservation of NF-κB family TFBS in the Biphasic genes

Table show average percentage of conserved NF-κB family TFBS in cross species comparisons for the Biphasic genes. For gene marked blue we found conserved TFBS in all species, for genes in red we did not find any conserved TFBS. Average percentage is equal to ½ of the sum of the percentages of conserved NF-κB family TFBS when promoter sequence of either *Species1* or *Species2* are used as templates.

|  | **Human- Chimpanzee** | **Human -Cattle** | **Human - Mouse** | **Chimpanzee -Cattle** | **Chimpanzee-Mouse** | **Cattle-Mouse** |
| --- | --- | --- | --- | --- | --- | --- |
| CYB5A | 100.00 | 0.00 | 11.76 | 0.00 | 9.73 | 0.00 |
| AQP3 | 100.00 | 0.00 | 0.00 | 0.00 | 0.00 | 0.00 |
| PSMB9 | 91.38 | 47.96 | 11.78 | 34.85 | 12.50 | 32.20 |
| PSMB8 | 0.00 | 0.00 | 0.00 | 0.00 | 0.00 | 0.00 |
| IFI35 | 84.13 | 9.40 | 0.00 | 0.00 | 0.00 | 27.78 |
| MVP | No homolog | 0.00 | 0.00 | No homolog | No homolog | 0.00 |

## Supplemental Table 4 - ARE content in the NF-κB dependent genes.

Table shows content of all ARE classes in 3’UTR of genes in all 4 species analysed in this paper. For species other than human some 3’UTR sequences were missing. these genes were removed from the table. Gene name is presented in the first column, followed by Reference Sequence ID (RefSeqID) and colour coding depicts the Early (green), Middle (grey) and Late (red) genes group.

| **Human** | **RefSeqID** | **ARE III** | **ARE I** | **ARE II** |
| --- | --- | --- | --- | --- |
| *Late* |  |  |  |  |
| TAP1 | NM_000593 [3`UTR] | 2 | 1 | 1 |
| TAPBP | NM_003190 [3`UTR] | 8 | 3 | 1 |
| IL32 | NM_001012631 [3`UTR] | 0 | 0 | 0 |
| TRAF1 | NM_005658 [3`UTR] | 1 | 1 | 0 |
| TRAF3 | NM_003300 [3`UTR] | 2 | 3 | 0 |
| PTGES | NM_004878 [3`UTR] | 1 | 1 | 0 |
| ICAM1 | NM_000201 [3`UTR] | 1 | 2 | 0 |
| IL27RA | NM_004843 [3`UTR] | 3 | 0 | 0 |
| NFKB2 | NM_001077493 [3`UTR] | 1 | 1 | 0 |
| TRIM16 | NM_006470 [3`UTR] | 3 | 0 | 0 |
| TNIP1 | NM_006058 [3`UTR] | 1 | 0 | 0 |
| *Middle* | |  |  |  |
| BID | NM_001196 [3`UTR] | 6 | 2 | 0 |
| BIRC2 | NM_001166 [3`UTR] | 5 | 3 | 1 |
| TNFAIP2 | NM_006291 [3`UTR] | 2 | 0 | 0 |
| CFB | NM_001710 [3`UTR] | 1 | 0 | 0 |
| NFKBIE | NM_004556 [3`UTR] | 0 | 0 | 0 |
| BCL3 | NM_005178 [3`UTR] | 1 | 0 | 0 |
| TRAF2 | NM_021138 [3`UTR] | 1 | 0 | 0 |
| SOD2 | NM_000636 [3`UTR] | 2 | 2 | 0 |
| GCH1 | NM_000161 [3`UTR] | 13 | 11 | 1 |
| GFPT2 | NM_005110 [3`UTR] | 1 | 2 | 1 |
| KLRC3 | NM_002261 [3`UTR] | 4 | 3 | 0 |
| SDC4 | NM_002999 [3`UTR] | 10 | 3 | 0 |
| SLC7A2 | NM_001008539 [3`UTR] | 24 | 12 | 5 |
| CD83 | NM_001040280 [3`UTR] | 4 | 4 | 0 |
| IFNGR2 | NM_005534 [3`UTR] | 2 | 1 | 0 |
| ECE1 | NM_001113347 [3`UTR] | 7 | 3 | 1 |
| NFKB1 | NM_003998 [3`UTR] | 7 | 3 | 1 |
| RELB | NM_006509 [3`UTR] | 1 | 0 | 0 |
| *Early* |  |  |  |  |
| TNFAIP3 | NM_006290 [3`UTR] | 14 | 5 | 2 |
| IL8 | NM_000584 [3`UTR] | 16 | 9 | 3 |
| IL6 | NM_000600 [3`UTR] | 8 | 6 | 4 |
| TNF | NM_000594 [3`UTR] | 2 | 9 | 5 |
| CXCL1 | NM_001511 [3`UTR] | 6 | 5 | 4 |
| CXCL3 | NM_002090 [3`UTR] | 6 | 7 | 3 |
| CXCL2 | NM_002089 [3`UTR] | 5 | 10 | 9 |
| CCL20 | NM_001130046 [3`UTR] | 5 | 3 | 1 |
| EFNA1 | NM_004428 [3`UTR] | 2 | 0 | 0 |
| NFKBIA | NM_020529 [3`UTR] | 6 | 3 | 3 |
| PTGS2 | NM_000963 [3`UTR] | 27 | 22 | 11 |
| REL | NM_002908 [3`UTR] | 3 | 2 | 1 |
| IRF1 | NM_002198 [3`UTR] | 9 | 4 | 1 |
| PLAU | NM_001145031 [3`UTR] | 5 | 2 | 2 |

| **Chimpanzee** | **RefSeqID** | **ARE III** | **ARE I** | **ARE II** |
| --- | --- | --- | --- | --- |
| *Late* |  |  |  |  |
| TAPBP | XM_001170532 [3`UTR] | 13 | 1 | 3 |
| TRAF1 | XM_001158357 [3`UTR] | 3 | 0 | 1 |
| TRAF3 | XM_001164813 [3`UTR] | 10 | 1 | 5 |
| PTGES | XM_528441 [3`UTR] | 1 | 0 | 1 |
| ICAM1 | NM_001009946 [3`UTR] | 0 | 0 | 0 |
| IL27RA | XM_512969 [3`UTR] | 0 | 0 | 0 |
| *Midle* | |  |  |  |
| BID | XM_514966 [3`UTR] | 19 | 2 | 6 |
| BIRC2 | XM_001152344 [3`UTR] | 5 | 1 | 3 |
| TNFAIP2 | XM_001134933 [3`UTR] | 0 | 0 | 0 |
| CFB | NM_001009169 [3`UTR] | 0 | 0 | 0 |
| NFKBIE | XM_518507 [3`UTR] | 1 | 0 | 0 |
| GCH1 | XM_001161570 [3`UTR] | 11 | 1 | 6 |
| GFPT2 | XM_518158 [3`UTR] | 2 | 1 | 2 |
| KLRC3 | NM_001009017 [3`UTR] | 0 | 0 | 0 |
| SDC4 | XM_525337 [3`UTR] | 13 | 0 | 3 |
| SLC7A2 | XM_001142615 [3`UTR] | 1 | 1 | 1 |
| CD83 | XM_518248 [3`UTR] | 6 | 0 | 4 |
| IFNGR2 | XM_525461 [3`UTR] | 2 | 0 | 1 |
| ECE1 | XM_001162689 [3`UTR] | 6 | 1 | 3 |
| NFKB1 | XM_001168718 [3`UTR] | 7 | 1 | 3 |
| RELB | XM_512742 [3`UTR] | 0 | 0 | 0 |
| *Early* |  |  |  |  |
| TNFAIP3 | XM_527515 [3`UTR] | 18 | 2 | 5 |
| IL8 | XM_001156375 [3`UTR] | 18 | 2 | 9 |
| IL6 | XM_001154511 [3`UTR] | 9 | 4 | 6 |
| TNF | NM_001045511 [3`UTR] | 0 | 0 | 0 |
| CXCL1 | XM_001156094 [3`UTR] | 9 | 4 | 6 |
| CXCL3 | XM_001155685 [3`UTR] | 13 | 4 | 8 |
| CXCL2 | XM_001155614 [3`UTR] | 11 | 9 | 11 |
| CCL20 | XM_516133 [3`UTR] | 6 | 1 | 4 |
| EFNA1 | XM_001141980 [3`UTR] | 0 | 0 | 0 |
| NFKBIA | XM_522823 [3`UTR] | 1 | 0 | 0 |
| PTGS2 | XM_524999 [3`UTR] | 31 | 11 | 22 |
| REL | XM_001159382 [3`UTR] | 5 | 1 | 1 |
| IRF1 | XM_001150404 [3`UTR] | 4 | 1 | 3 |

| **Cattle** | **RefSeqID** | **ARE III** | **ARE I** | **ARE II** |
| --- | --- | --- | --- | --- |
| *Late* |  |  |  |  |
| TAP1 | NM_001098058 [3`UTR] | 0 | 0 | 0 |
| TAPBP | NM_001045885 [3`UTR] | 3 | 4 | 0 |
| TRAF1 | NM_001192801 [3`UTR] | 0 | 0 | 0 |
| TRAF3 | XM_582595 [3`UTR] | 1 | 2 | 0 |
| PTGES | NM_174443 [3`UTR] | 1 | 0 | 0 |
| ICAM1 | NM_174348 [3`UTR] | 1 | 2 | 1 |
| IL27RA | NM_001098028 [3`UTR] | 2 | 0 | 0 |
| NFKB2 | NM_001102101 [3`UTR] | 1 | 1 | 0 |
| TNIP | NM_001024554 [3`UTR] | 1 | 0 | 0 |
| *Middle* |  |  |  |  |
| BID | NM_001075446 [3`UTR] | 1 | 0 | 0 |
| TNFAIP2 | XM_614791 [3`UTR] | 0 | 0 | 0 |
| CFB | NM_001040526 [3`UTR] | 1 | 0 | 0 |
| NFKBIE | NM_001130746 [3`UTR] | 2 | 0 | 0 |
| BCL3 | XM_610447 [3`UTR] | 0 | 1 | 0 |
| TRAF2 | XM_869007 [3`UTR] | 1 | 0 | 0 |
| SOD2 | NM_201527 [3`UTR] | 5 | 2 | 1 |
| GCH1 | XM_001251704 [3`UTR] | 5 | 4 | 2 |
| GFPT2 | NM_001076883 [3`UTR] | 2 | 1 | 1 |
| SDC4 | XM_584869 [3`UTR] | 8 | 4 | 0 |
| SLC7A2 | XM_865568 [3`UTR] | 0 | 0 | 0 |
| CD83 | NM_001046590 [3`UTR] | 4 | 2 | 0 |
| IFNGR2 | XM_592804 [3`UTR] | 3 | 1 | 0 |
| ECE1 | NM_181009 [3`UTR] | 4 | 2 | 1 |
| NFKB1 | NM_001076409 [3`UTR] | 6 | 2 | 1 |
| RELB | XM_600955 [3`UTR] | 0 | 0 | 0 |
| *Early* |  |  |  |  |
| TNFAIP3 | NM_001192170 [3`UTR] | 0 | 0 | 0 |
| IL8 | NM_173925 [3`UTR] | 13 | 11 | 6 |
| IL6 | NM_173923 [3`UTR] | 9 | 6 | 4 |
| TNFAIP3 | NM_173966 [3`UTR] | 4 | 10 | 6 |
| CXCL2 | NM_001046513 [3`UTR] | 6 | 7 | 4 |
| CCL20 | NM_174263 [3`UTR] | 0 | 0 | 0 |
| EFNA1 | NM_001034292 [3`UTR] | 3 | 1 | 0 |
| NFKBIA | NM_001045868 [3`UTR] | 7 | 2 | 2 |
| PTGS2 | NM_174445 [3`UTR] | 9 | 11 | 7 |
| REL | NM_001192970 [3`UTR] | 0 | 0 | 0 |
| IRF1 | NM_177432 [3`UTR] | 5 | 3 | 1 |
| PLAU | NM_174147 [3`UTR] | 2 | 2 | 2 |

| **Mouse** | **RefSeqID** | **ARE III** | **ARE I** | **ARE II** |
| --- | --- | --- | --- | --- |
| *Late* |  |  |  |  |
| TAP1 | NM_001161730 [3`UTR] | 0 | 1 | 0 |
| TAPBP | NM_009318 [3`UTR] | 3 | 0 | 0 |
| TRAF1 | NM_009421 [3`UTR] | 0 | 1 | 0 |
| TRAF3 | NM_011632 [3`UTR] | 5 | 3 | 1 |
| PTGES | NM_022415 [3`UTR] | 3 | 0 | 0 |
| ICAM1 | NM_010493 [3`UTR] | 1 | 4 | 0 |
| IL27RA | NM_016671 [3`UTR] | 2 | 0 | 0 |
| NFKB2 | NM_019408 [3`UTR] | 0 | 1 | 1 |
| TRIM16 | NM_053169 [3`UTR] | 4 | 3 | 1 |
| TNIP1 | NM_021327 [3`UTR] | 1 | 0 | 0 |
| *Middle* |  |  |  |  |
| BID | NM_007544 [3`UTR] | 2 | 0 | 0 |
| BIRC2 | NM_007465 [3`UTR] | 5 | 1 | 0 |
| TNFAIP2 | NM_009396 [3`UTR] | 3 | 2 | 0 |
| CFB | NM_008198 [3`UTR] | 1 | 0 | 0 |
| NFKBIE | NM_008690 [3`UTR] | 1 | 0 | 0 |
| BCL3 | NM_033601 [3`UTR] | 1 | 0 | 0 |
| TRAF2 | NM_009422 [3`UTR] | 0 | 1 | 0 |
| SOD2 | NM_013671 | - | - | - |
| GCH1 | NM_008102 [3`UTR] | 5 | 7 | 1 |
| GFPT2 | NM_013529 [3`UTR] | 1 | 2 | 1 |
| KLRC3 | NM_021378 [3`UTR] | 0 | 0 | 0 |
| SDC4 | NM_011521 [3`UTR] | 6 | 1 | 0 |
| SLC7A2 | NM_007514 [3`UTR] | 17 | 12 | 3 |
| CD83 | NM_009856 [3`UTR] | 3 | 2 | 0 |
| IFNGR2 | NM_008338 [3`UTR] | 3 | 2 | 0 |
| ECE1 | NM_199307 [3`UTR] | 8 | 3 | 1 |
| NFKB1 | NM_008689 [3`UTR] | 9 | 4 | 1 |
| RELB | NM_009046 [3`UTR] | 2 | 0 | 0 |
| *Early* |  |  |  |  |
| TNFAIP3 | NM_001166402 [3`UTR] | 6 | 5 | 3 |
| IL6 | NM_031168 [3`UTR] | 7 | 5 | 4 |
| TNF | NM_013693 [3`UTR] | 3 | 8 | 5 |
| CXCL1 | NM_008176 [3`UTR] | 8 | 7 | 3 |
| CXCL3 | NM_203320 [3`UTR] | 7 | 5 | 4 |
| CXCL2 | NM_009140 [3`UTR] | 7 | 11 | 5 |
| CCL20 | NM_001159738 [3`UTR] | 3 | 5 | 3 |
| EFNA1 | NM_010107 [3`UTR] | 2 | 0 | 0 |
| NFKBIA | NM_010907 [3`UTR] | 6 | 2 | 2 |
| PTGS2 | NM_011198 [3`UTR] | 16 | 12 | 7 |
| REL | NM_009044 [3`UTR] | 1 | 2 | 0 |
| IRF1 | NM_001159396 [3`UTR] | 4 | 3 | 1 |
| PLAU | NM_008873 [3`UTR] | 5 | 2 | 2 |
