# Supplementary figures and images for: The dependence of expression of NF-κB-dependent genes: statistics and evolutionary conservation of control sequences in the promoter and in the 3′ UTR
Source: BMC Genomics. 2012 May 11;13:182. doi: 10.1186/1471-2164-13-182 (PMC3488004; doi:10.1186/1471-2164-13-182)

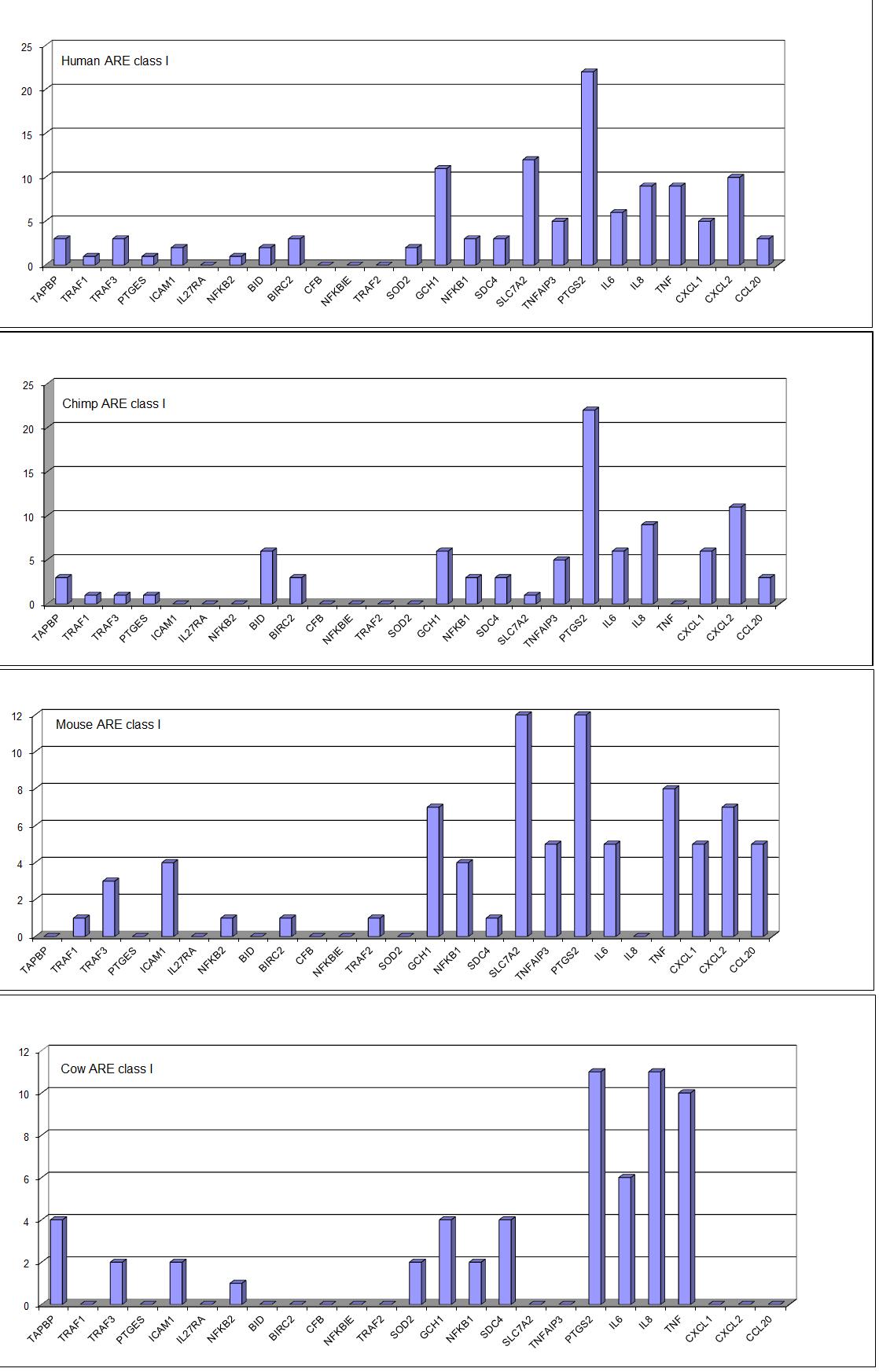

Supplement: Additional file 2 — Figure 5. [file 1471-2164-13-182-S2.jpeg]

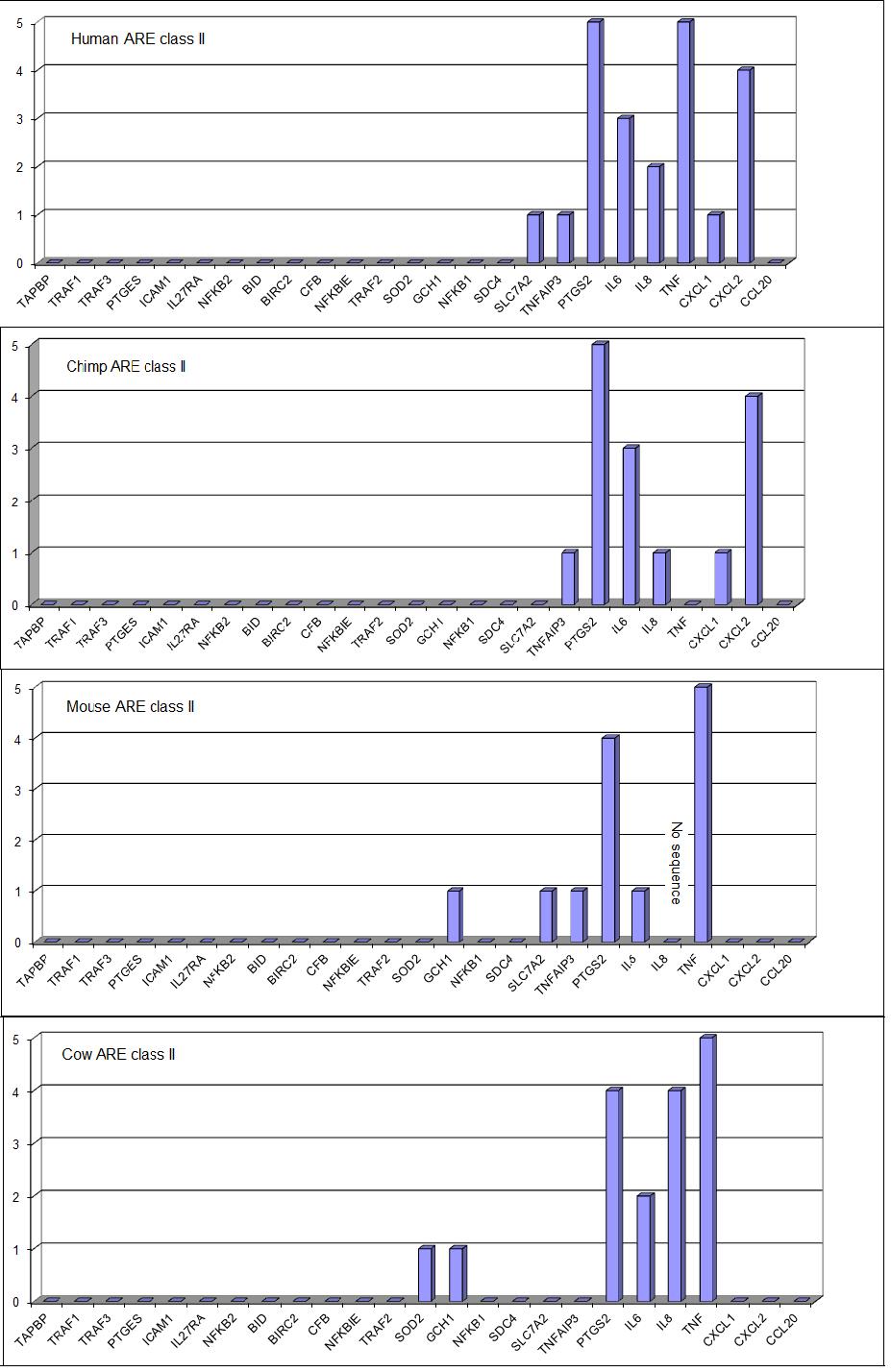

Supplement: Additional file 3 — Figure 6. [file 1471-2164-13-182-S3.jpeg]

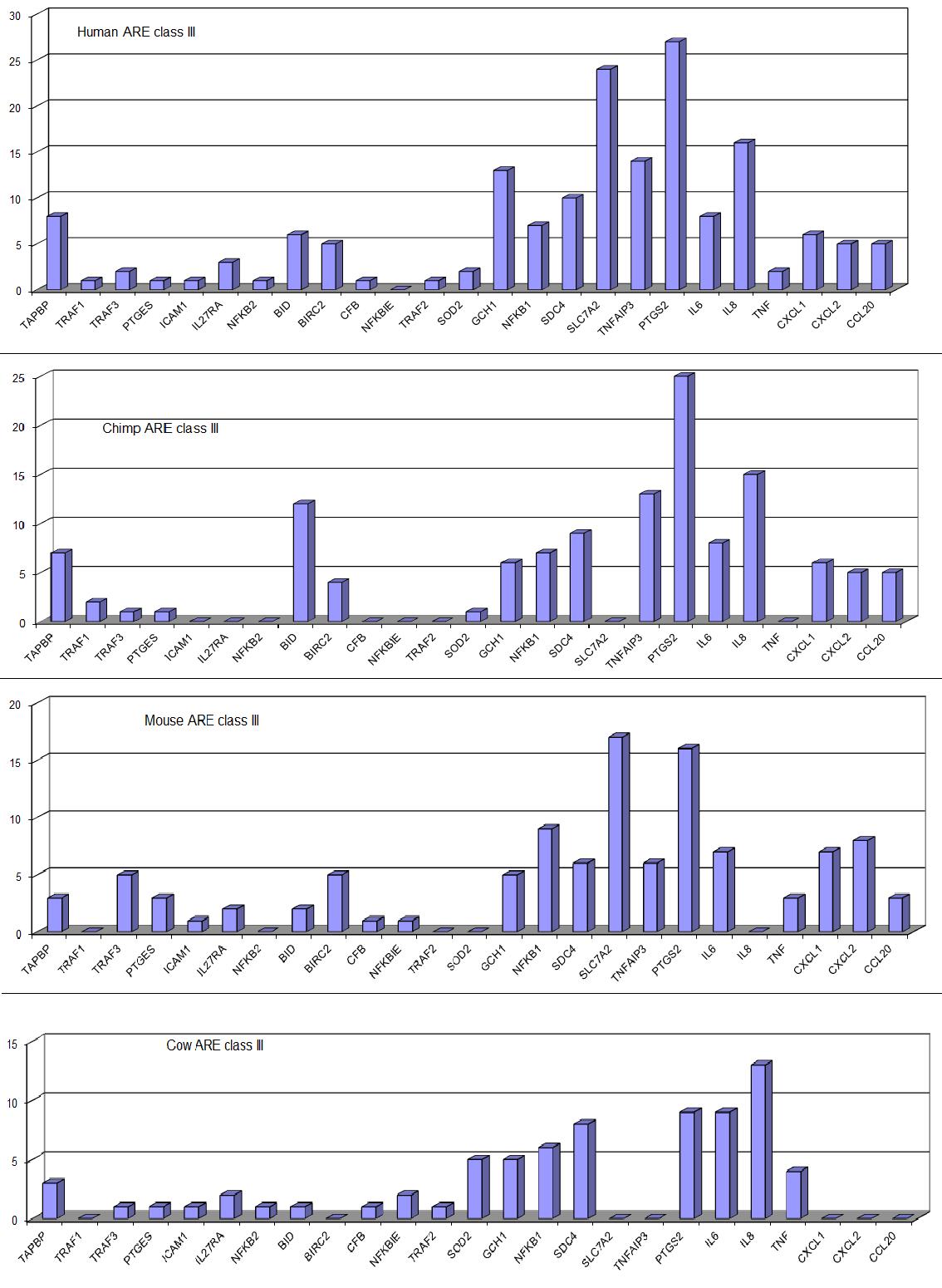

Supplement: Additional file 4 — Figure 7. [file 1471-2164-13-182-S4.jpeg]
